# Surgical explantation of quaternate transcatheter aortic valve replacement bioprostheses: a case report

**DOI:** 10.1186/s13019-022-01973-w

**Published:** 2022-10-17

**Authors:** Mengya Liang, Bohao Jian, Guangxian Chen, Zhongkai Wu

**Affiliations:** Department of Cardiac Surgery, The First Affiliated Hospital, Sun Yat-Sen University, #58, Zhongshan 2nd Road, Guangzhou, 510080 China

**Keywords:** Transcatheter aortic valve replacement, Self-expanding prosthesis, Paravalvular leakage, Surgical explantation

## Abstract

**Background:**

Transcatheter aortic valve replacement (TAVR) is a growing less invasive surrogate for high-risk patients with aortic valve disease. Although the number of TAVR procedures is growing rapidly, TAVR prosthesis surgical explantation are rare procedures but increasing in frequency.

**Case presentation:**

We herein presented a case of 68-year-old male who underwent quaternate TAVR bioprostheses implantation. Three months later, his symptoms deteriorated due to aggravated paravalvular leakage and severe mitral regurgitation. A challenging surgical explantation procedure was therefore performed. During the surgery, lethal penetrations of aortic wall due to migration of these devices were found and four bioprostheses were integrally explanted. The native calcified aortic leaflet was removed and replaced with a 23 mm. The impaired segment of ascending aorta was replaced with a Dacron graft afterwards.

**Conclusions:**

In summary, we presented a surgical case of explantation of four TAVR Bioprostheses, which is so far the maximum number of surgical-explant devices ever reported. This extreme individual case aggregates our technical experiences with this unique category of patients and raise the concern of the best initial valve strategy for relatively younger patients with severe aortic valve stenosis.

**Supplementary Information:**

The online version contains supplementary material available at 10.1186/s13019-022-01973-w.

## Background

Transcatheter aortic valve replacement (TAVR) is a growing less invasive surrogate for high-risk patients with aortic valve disease. The favorable outcomes and improved long-term survival of TAVR has been well-established by a series of clinical studies [1]. As the indication continues to expand in the lower-risk patients, a rapid augmentation of TAVR population is expected. TAVR prosthesis surgical explantation are rare procedures but increasing in frequency [2]. Due to the diversity of the causes and relatively high mortality, TAVR explant is still regarded as one of the most challenging procedures to cardiac surgeons. We herein reported a rare surgical case of explantation of quaternate TAVR bioprostheses.

## Case presentation

A 68-year-old male patient was referred to our institution with complaints of refractory chest pain and shortness of breath. His past medical history was notable for severe stenosis due to bicuspid aortic valve. A TAVR procedure with four bioprostheses was performed through femoral route in other institute three months before. The preoperative echocardiography demonstrated a reduced LVEF (37%) and he had stage four CKD. During the previous operation the first device (26 mm Venus A self-expanding bioprosthesis, VENUS MEDTECH, China) migrated from the anulus to the ascending aorta soon after deployment. The hemodynamics deteriorated rapidly and attempt of repositioning was failed. Another 26 mm prosthesis was immediately prepped and positioned with the lower mark at 5 mm below the bottom of non-coronary sinus. However, a moderate paravalvular leakage (PVL) was noticed after the deployment of the second prosthesis. A third 26 mm and a fourth 23 mm prosthesis were deployed inside the gap between the first and second device, with the goal to eliminate the leakage. The patients were then transferred to ICU with a residual mild PVL and was discharged at post-op day 7. He was doing well for the first postoperative months until the symptoms deteriorated in the next month.

**Fig. 1:**
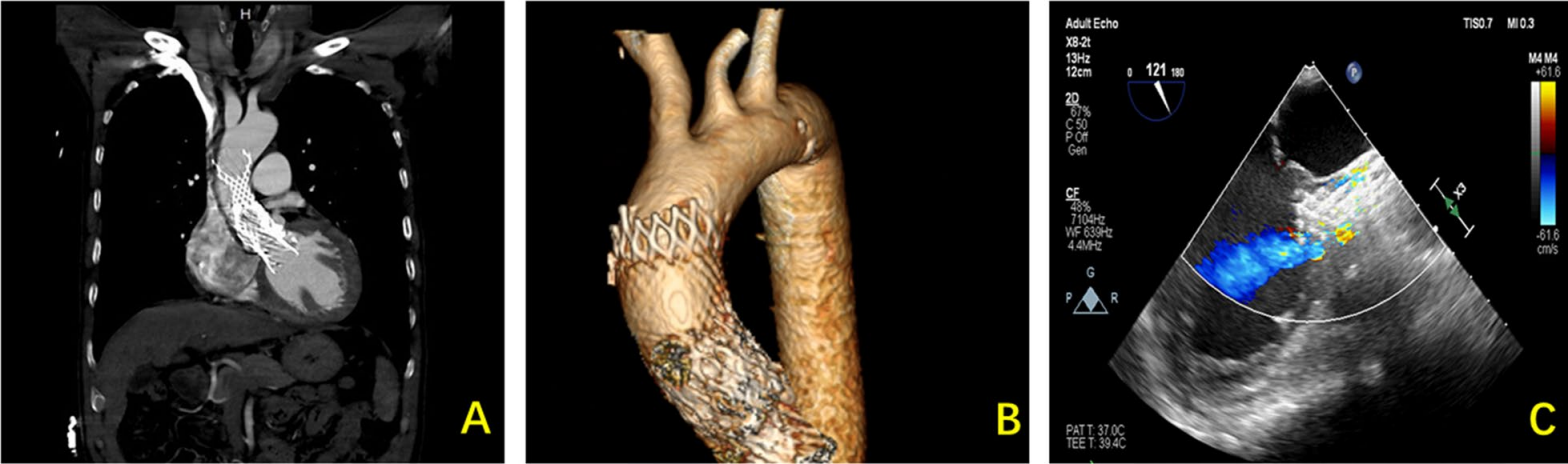
Preoperative Images of TAVR prosthesis migration in a male patient who underwent a previous TAVR procedure with four bioprostheses. **A** High density shadows of the devices were overlapping inside the ascending aorta. **B** The crown frame of the upper device was identified to protrude outside of the aortic wall. **C** A severe paravalvular leakage was identified by preoperative transesophageal echocardiography

The CTA demonstrated four high density shadow were located inside the ascending aorta (Fig. 1A), crown frame of the upper device was identified to protrude outside of the aortic wall (Fig. 1B). Additionally, the echocardiography showed that the PVL aggravated and severe mitral regurgitation developed due to the deep deployment of the TAVR device (Fig. 1C).

**Fig. 2:**
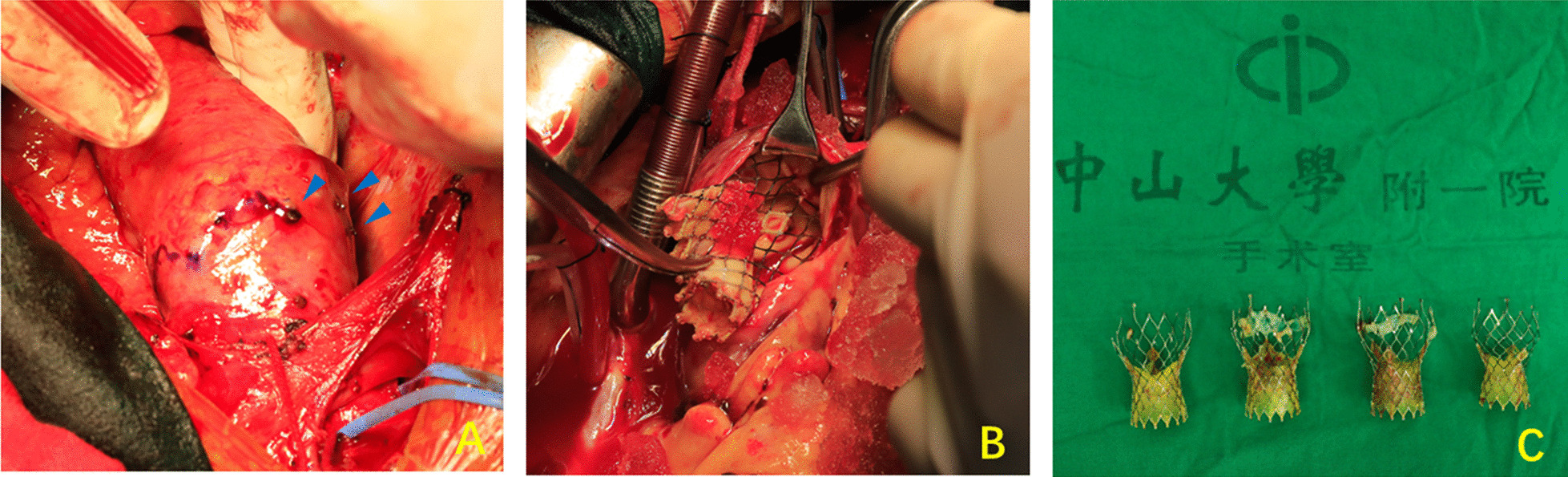
Intraoperative image of surgical explantation of TAVR prostheses. **A** The penetrations of aortic wall by the bioprosthesis crown were confirmed (blue arrows). **B** Circumferential neoendotheliazation was presented most prominently at the outermost device and the other three devices were sited inside the left ventricular outflow tract and overlapped. **C** Four TAVR bioprostheses were integrally explanted




A surgical explantation was performed soon after the admission (see Video). The femoral artery and vein were cannulated to establish the cardio-pulmonary bypass before median sternotomy, right axillary artery was cannulated for cerebral perfusion. Hematopericardium was discovered after the pericardium was opened. Penetrations of aortic wall by knobs of the bioprosthesis crown was confirmed (Fig. 2A). Retrograde cardioplegia was delivered because the ostias of coronary arteries were inaccessible under such circumstance. A safe cross-clamp site was dissected between the anonymous artery and left common carotid artery. After the heart arrest, a transverse aortotomy was performed and the prostheses were exposed. The first (upper) device was stuck inside the ascending aorta and the other three devices were overlapped and sited inside the left ventricular outflow tract. Circumferential neoendotheliazation was presented most prominently at the outermost prosthesis (Fig. 2B), careful dissection was made to avoid injuries to the aortic wall. The valve scaffolds were compressed and folded with the aid of Kocher clamps, thereafter Four bioprostheses were integrally explanted (Fig. 2C). The native calcified aortic leaflet was then removed and inspection was made to confirm the normal functioning of mitral valve. A 23 mm bioprosthesis (Perimount Magna, Edwards Lifescience) was then implanted. Afterward, the impaired segment of ascending aorta was replaced with a Dacron graft and the patient was weaned from CPB. He recovered well and was discharged on post-op day 17.

## Discussion and conclusions

An analysis from the Society of Thoracic Surgeons Adult Cardiac Surgery Database have described surgical explants with an incidence of 0.4% to 1% of index TAVR [2]. The indications of TAVR explant were often related to procedure-related failure (35%), paravalvular leak (28%), need for other cardiac surgery (26%), endocarditis (13%) and structural valve degeneration (11%) et al [2]. Surgical mortality of these procedures rises to triple times comparing to that at the first index TAVR procedure and at least two-thirds of patients required concomitant cardiac surgical procedures [2]. however, due to paucity of surgical reports and lack of long-term follow-up, surgeons may encounter multiple uncertainties and difficult decision-makings when treating these patients.

In reference to previous reports [3], several issues must be addressed preoperatively and some recommended techniques is helpful in explantation: (1) The neoendothelialization is commonly seen if TAVR valves are older than 1 year, otherwise they are overlapped when more than one device are implanted, careful endarterectomy is performed to separate the device from the aortic intima. Afterwards careful inspection of the aortic wall is warranted to decide whether a concomitant graft replacement should be performed, which occurred in 25.6% of the population. (2) Retrograde cardioplegia is the first choice of cardioplegic delivery because the frame of prosthesis often overlaps the coronary ostia and the sinuses are occupied by the rolled up native leaflet. (3) Explantation of a relatively young TAVR valve does not require any special technique and equipment regardless of the valve type, also cooling of the alloy frame is not mandatory [4]. Nevertheless, surgical explantation of multiple devices requires significant planning and preparation to guarantee a successful procedure.

Although the current surgical-explant is an extreme individual case, it raises a concern for the appropriateness of TAVR as a first valve strategy in younger, healthier patients. The frequency of TAVR-explant is estimated to be at least as common as redo TAVR [4]. Additionally, concomitant aortic procedures were performed in 33% of patients underwent TAVR-explant, which inevitably increase the mortality rates. With the improvement of surgical outcomes of SAVR, we believe discreet reconsideration of expansion of TAVR indication to younger patients is necessary.

In addition, one concern raised by these cases is the migration probability of the self-expanding prosthesis. In the light of TAVR-explant operative data, 66% explanted devices are self-expanding prosthesis whereas 26% devices are balloon-expandable devices. It seems that the self-expanding prosthesis is more likely to migrate when it is implanted in a higher position in heavily calcified BAV [4]. If it really happened, after repositioning the failed valve, intense follow-up should be arranged for these patients, because further migration of the displaced device will result in lethal penetration of aorta. Additionally, the surgical explantation of self-expanding prosthesis is reported to have higher incidence of conduit replacement of aorta than other devices due to its unique shape characteristics. Another concern is the higher transaortic gradient and higher risk of pacemaker implantation of self-expanding prosthesis compared with balloon-expandable devices [5]. However, despite all these concerns, increasing amount of TAVR population and the considerable proportion of self-expanding valves explantation highlight the importance of optimal therapy for complicated cases.

In summary, we presented a surgical case of explantation of four TAVR Bioprostheses, which is so far the maximum number of surgical-explant devices ever reported. This extreme individual case aggregates our technical experiences with this unique category of patients and raise the concern of the best initial valve strategy for relatively younger patients with severe aortic valve stenosis.

## Data Availability

Not applicable.

## Abbreviations

TAVR: Transcatheter aortic valve replacement
PVL: Paravalvular leakage
ICU: Intensive care unit
CTA: Computed tomography angiography
CPB: Cardiopulmonary bypass
SAVR: Surgical aortic valve replacement
BAV: Bicuspid aortic valve

## References

[1] Siontis GCM, Overtchouk P, Cahill TJ (2019). Transcatheter aortic valve implantation vs. surgical aortic valve replacement for treatment of symptomatic severe aortic stenosis: an updated meta-analysis. Eur Heart J.

[2] Fukuhara S, Brescia AA, Shiomi S (2021). Surgical explantation of transcatheter aortic bioprostheses: results and clinical implications. J Thorac Cardiovasc Surg.

[3] Mangi AA, Ramchandani M, Reardon M (2018). Surgical removal and replacement of chronically implanted transcatheter aortic prostheses: how I teach it. Ann Thorac Surg.

[4] Fukuhara S, Brescia AA, Deeb GM (2020). Surgical explantation of transcatheter aortic bioprostheses: an analysis from the Society of Thoracic Surgeons Database. Circulation.

[5] Ou-Yang WB (2022). Propensity-matched comparison of balloon-expandable and self-expanding valves for transcatheter aortic valve replacement in a Chinese population. Ann Transl Med.

